# Of the Point of Departure in the Spinal Cord, of the Especial Conductors Which Contract the Muscles in Epileptiform Convulsions

**Published:** 1870-03

**Authors:** 


### OF THE POINT OF DEPARTURE IN THE SPINAL CORD, OF THE ESPECIAL CONDUCTORS WHICH CONTRACT THE MUSCLES IN EPILEPTIFORM CONVULSIONS.

By DR. BROWN-SÉQUARD.

Translated for the EXAMINER, from *Archives de Physiologie et Normale et Pathologique.*

I have shown in a previous article (*Archives*, Sept., 1869, p. 612), that the conductors controlling the voluntary movements are distinct from those which contract the muscles of the abdominal members in epileptic attacks. The facts I am about to relate affor l additional proof of the accuracy of this conclusion; but they show, moreover, that the conductors capable of causing some convulsions originate in another portion of the spinal cord, than where the conductors of the voluntary movements are found.

If an incision is confined to one of the two posterior columns of the spinal nerve, at the level of the last dorsal vertibræ, of a Guinea-pig, when the animal becoming epileptic has an attack, some convulsions take place in the abdominal member of the injured side, and are more violent and prolonged there than elsewhere.

If a deeper incision is made in the posterior part of the spinal cord, comprizing, in addition to one of the posterior columns, a portion of the gray substance and of the lateral column of the same side, it is seen, after the epilepsy has reached its greatest intensity, that the convulsions disappear almost entirely in the abdominal muscles of the corresponding side, while the voluntary movement remains there without perceptible diminution.

If a still greater lesion is made, affecting nearly the entire lateral half of the spinal cord, the voluntary movement subsides considerably in the posterior member of the same side, and seems even, after a time, to disappear altogether, the convulsions disappearing there also, be it understood, completely.

If, upon the same animal two transverse sections are made, at the same level as in the preceding experiments, one upon one of the posterior columns, the other upon the anterior column and the anterior gray cornu of the same side, there will be seen (in cases where the remainder of the cord, of this side, is not perceptibly altered *), in the paroxysms of epilepsy, some quite strong convulsions, in the abdominal muscles of the injured side, while the voluntary movements are considerably diminished there.

Finally, if, as I have two examples before me, nearly one entire side, and, also, a great part of the other, half of the spinal cord is severed, leaving, meanwhile, among other intact parts, a considerable portion of the lateral cord,† it will be ascertained that the convulsions disappear in the abdominal muscles, on the side of the more extensive injury, while they occur with violence in the other abdominal muscles, both, however, retaining the voluntary movement in nearly the same degree.

These facts concur to establish, in an evident manner—

1st. That the conductors capable of causing some convulsions are distinct from those which control the voluntary movements.

2d. That the place of exit, of the first, is different from that of the second, in the spinal cord, and that the first are mainly situated in the lateral cord, and, perhaps, in the neighboring

* I have only succeeded once in making this experiment in a satisfactory manner; but the success, in this case, has been as complete and decisive as possible.

† Autopsy not having been made of the two animals subjected to this last experiment, I cannot indicate precisely the injury. Anæsthesia, which appeared complete, existed in the two abdominal members; the voluntary movements in these members are diminished, but regular, and the temperature preserves its normal degree.

gray substance, while the second are located principally in the gray and white anterior parts of this nervous centre.

